# High-Rate Anaerobic Digestion of Waste Activated Sludge by Integration of Electro-Fenton Process

**DOI:** 10.3390/molecules25030626

**Published:** 2020-01-31

**Authors:** Emna Feki, Audrey Battimelli, Sami Sayadi, Abdelhafidh Dhouib, Sonia Khoufi

**Affiliations:** 1 Laboratory of Environmental Bioprocesses, Centre of Biotechnology of Sfax, BP 1177, Sfax 3018, Tunisia; emnafeki2010@hotmail.fr (E.F.); abdelhfidh.douib@cbs.rnrt.tn (A.D.); 2 INRAE, Université de Montpellier, Laboratoire de Biotechnologie de l’Environnement, 102 avenue des Etangs, 11100 Narbonne, France; 3 Center for Sustainable Development, College of Arts and Sciences, Qatar University, Doha 2713, Qatar

**Keywords:** anaerobic digestion, biogas yield, waste activated sludge, electro-Fenton, disintegration, dewaterability

## Abstract

Anaerobic digestion (AD), being the most effective treatment method of waste activated sludge (WAS), allows for safe disposal. The present study deals with the electro-Fenton (EF) pretreatment for enhancing the WAS biogas potential with low-cost iron electrodes. The effect of pretreatment on the physicochemical characteristics of sludge was assessed. Following EF pretreatment, the pH, conductivity, soluble chemical oxygen demand (SCOD), and volatile fatty acids (VFA) increased to 7.5, 13.72 mS/cm, 4.1 g/L, and 925 mg/L, respectively. Capillary suction time (CST) analysis highlighted the dewaterability effect of EF on WAS, as demonstrated by the decrease in CST from 429 to 180 s following 30 min of pretreatment. Batch digestion assays presented an increase in the biogas yield to 0.135 L/g volatile solids (VS) after 60 min of EF pretreatment in comparison to raw sludge (0.08 L/g VS). Production of biogas was also found to improve during semi-continuous fermentation of EF-pretreated sludge conducted in a lab-scale reactor. In comparison to raw sludge, EF-pretreated sludge produced the highest biogas yield (0.81 L biogas/g VS) with a high COD removal rate, reaching 96.6% at an organic loading rate (OLR) of 2.5 g VS/L. d. Results revealed that the EF process could be an effective WAS disintegration method with maximum recovery of bioenergy during AD.

## 1. Introduction

Municipal wastewater treatment holds an important role in environmental concerns since wastewater discharges have greatly evolved in quantity and quality during recent decades. Activated sludge process is the main effective method used for treatment of municipal wastewater and thus leads to the production of large quantities of waste activated sludge (WAS). This residue, composed of cellular biomass (20–40%), refractory organic matter (40–60%), and mineral matter (10–30%), contains various minerals, organic micropollutants, and pathogenic organisms which can be potentially harmful for the environment and present genuine management problems [1]. With such environmental and economic concerns, there is growing interest focusing on the reduction in volume and stabilization of WAS [2].

Nowadays, thanks to its environmental and economic benefits, anaerobic digestion (AD) is considered to be the best option for managing WAS [3]. It is widely used as the most cost-effective way for stabilization, pathogen removal, and energy recovery. In addition, AD favors the reduction of sludge volume while producing clean energy in the form of biogas [4]. This biogas can be upgraded for direct utilization as transport fuel or injection into the gas grid. It can also be utilized in combined heat and power systems for providing electricity and heat to the wastewater treatment plant (WWTP) or for export in cases of overproduction. Any improvement in the AD efficiency should therefore lead to a further reduction in sludge volume for transport and disposal. It is likely that the biogas yield will increase, hence producing a greater amount of renewable energy and resulting in higher environmental performance and savings for the plant [5]. AD comprises a series of reactions including hydrolysis, acidogenesis, acetogenesis, and methanogenesis. Hydrolysis is the rate-limiting step for the AD of activated sludge because most organic matter present in WAS is enclosed in cell walls and membranes that protect the intracellular components [6]. The cells are shielded against osmotic lysis thanks to the semi rigid structure of the cell envelope [7]. Sludge retention time (SRT) or hydraulic retention time (HRT) are believed to be key parameters in sludge AD [8]. Various studies focus on the effect of HRT on reactor performance such as biogas production and volatile solid destruction [9]. It has often been demonstrated that a high HRT is necessary (20–30 days) to obtain a 30–50% degradation efficiency of organic solids [10]. Cell lysis has been referred as a possible method for releasing intracellular organics and increasing the rate and efficiency of the digestion process [6]. Indeed, this is possible when extracellular polymeric substances (EPS) become more bioavailable [11].

Recently, different pretreatments have been examined for improving the physicochemical characteristics of organic waste and consequently the performance of anaerobic digestion [12]. During several studies performed in half-scale and lab-scale plants, these methods, including chemical and thermal methods [12,13,14], as well as mechanical [15] and biological hydrolysis with enzymes [16,17], were investigated for sludge disintegration purposes. By increasing the digestion rate, biogas yields were maximized in smaller digesters while the HRTs decreased [11]. Currently, advanced oxidation processes (AOPs) are considered as valuable sludge pretreatments that might reduce hydraulic retention times and increase methane production rates [18,19]. These innovative technologies are widely used for the treatment of polluted waters. They apply the concept of producing hydroxyl radicals (HO·) which are capable of decomposing a number of organic substances via oxidation. AOPs include a series of powerful technologies: photo-catalysis, Fenton reaction, photo-Fenton, etc. Recently, researchers have focused on the disintegration of WAS by electro-Fenton (EF), although few studies have evaluated the performance of EF pretreatment on WAS anaerobic biodegradability as well as its biogas potential [19,20]. The electro-Fenton process is an advanced electrochemical oxidation process that comprises several steps. It involves electrochemical reactions that generate the reagents used for the Fenton reaction in situ. The generated reagents depend on the solution and on the nature of the electrodes. Generally, with an inert electrode, oxidation occurs via the hydroxyl radicals formed during the electrolysis of water [19].

Here, the EF process was investigated as a pretreatment step for improving the AD of activated sludge and its biogas potential. Low-cost iron electrodes were employed during EF pretreatment for in situ generation of Fe^2+^. The effect of this pretreatment on the solubilization and dewaterability of flocs was investigated. Biochemical methane potential tests were conducted to optimize the pretreatment time in terms of maximum biogas yields. The performance of an anaerobic reactor fed with EF-pretreated sludge and operated under semi-continuous conditions was also assessed.

## 2. Results

### 2.1. Disintegration of Activated Sludge by Electro-Fenton Process

#### 2.1.1. Physicochemical Analysis

**Figure 1:**
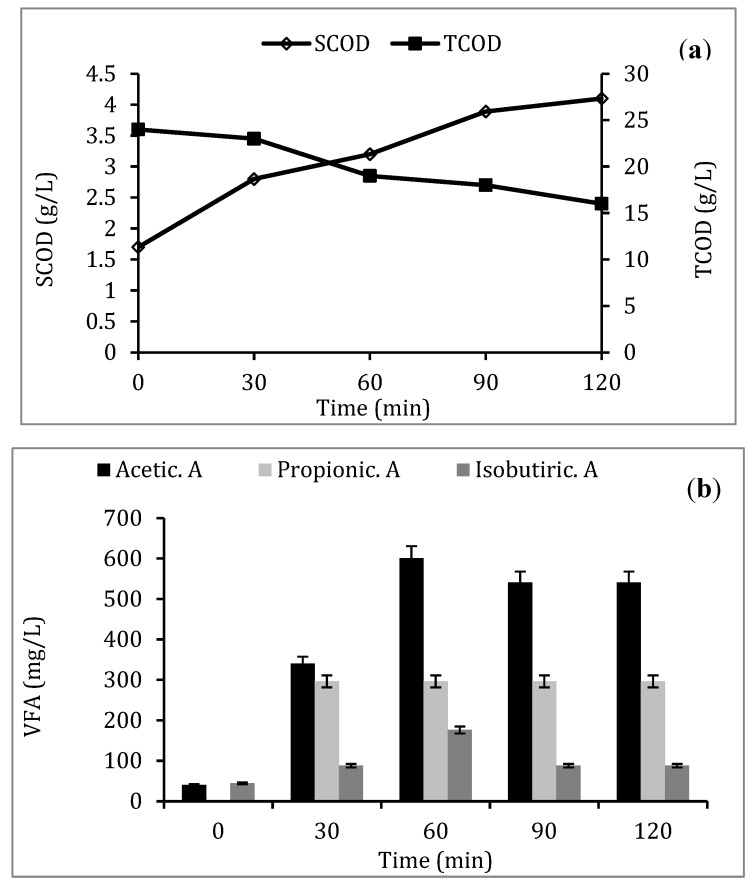

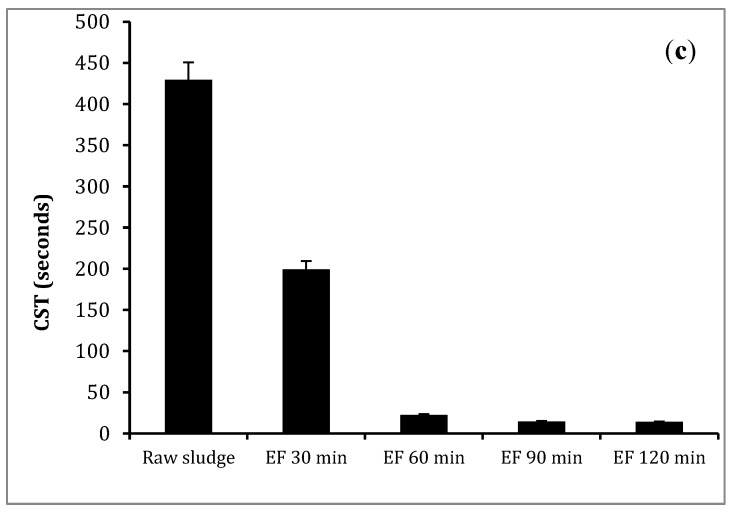
Evolution of soluble and total chemical oxygen demand (**a**), volatile fatty acid concentrations (**b**), and capillary suction time (**c**) during electro-Fenton (EF) pretreatment of WAS.

During the EF reaction, pH, conductivity, total and soluble COD, and VFA analyses were performed (Table 1). Sludge pH increased with treatment time and reached 7.5 after 2 h of reaction. At the same time, conductivity increased to 13.72 mS/cm. The soluble COD of raw sludge is about 1.7 g/L. By applying an EF reaction, a gradual increase was observed up to a value of 4.1 g/L after 120 min of treatment time (Figure 1a). Meanwhile, a decrease in total COD occurred during the treatment, thus indicating a partial mineralization of organic matter. The volatile fatty acids concentration during the EF reaction increased as a function of the treatment time (Figure 1b). In the case of raw sludge, the total concentration of VFA is about 84.3 mg/L. This concentration increased to 1100 mg/L after 1 h then decreased with treatment time (925 mg/L at 2 h) (Figure 1b).

#### 2.1.2. Capillary Suction Time (CST) Analysis

To identify the effect of EF treatment on sludge dewaterability, the capillary suction time (CST) of raw and pretreated sludge was determined. CST is a fast and reliable method for determining the filterability of activated sludge.

Figure 1c illustrates the decrease in elapsed time during filtration with increasing sludge pretreatment time. The CST decreased from 429 to 180 s by treating sludge by EF during 30 min. This decrease was even higher after 1 h, while dewaterability improved by about 95%.

#### 2.1.3. Fourier Transform Infrared Spectroscopy Analysis (FTIR)

**Figure 2:**
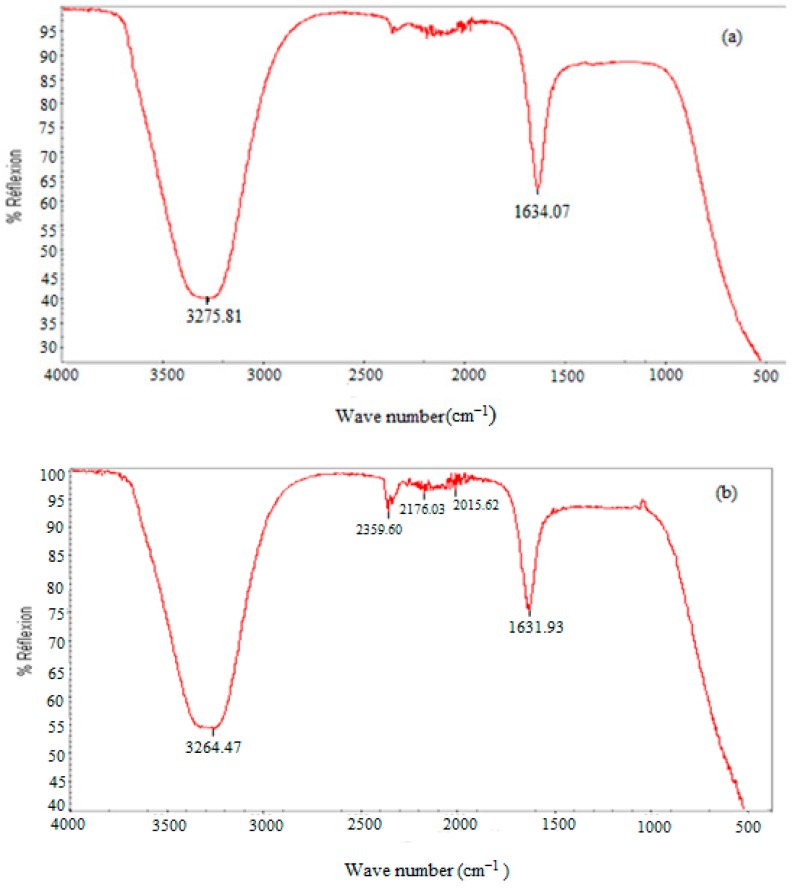
Spectroscopy of WAS before (**a**) and after (**b**) EF pretreatment.

The characterization of organic material in a sludge sample was performed by FTIR. Spectrums of the raw and EF-pretreated sludge presented in Figure 2 showed absorption bands related to biomass. Both samples are characterized by a strong peak around 3100–3500 cm^−1^. The second major band was identified between 1500 and 1700 cm^−1^ with a peak at 1634 and 1631 cm^−1^ for raw and pretreated sludge, respectively. The difference between the two spectrums is the fact that the intensity of the two major bands increased after EF pretreatment. New peaks also emerged in the pretreated sludge spectrum. The main absorption bands were thus observed in the range between 950 and 1100 cm^−1^. Other vibrations at 2015, 2176 and 2359 cm^−1^ between 2800 and 1900 cm^−1^ were also only observed in the pretreated sludge in comparison to raw WAS.

#### 2.1.4. Biogas Potential

**Figure 3:**
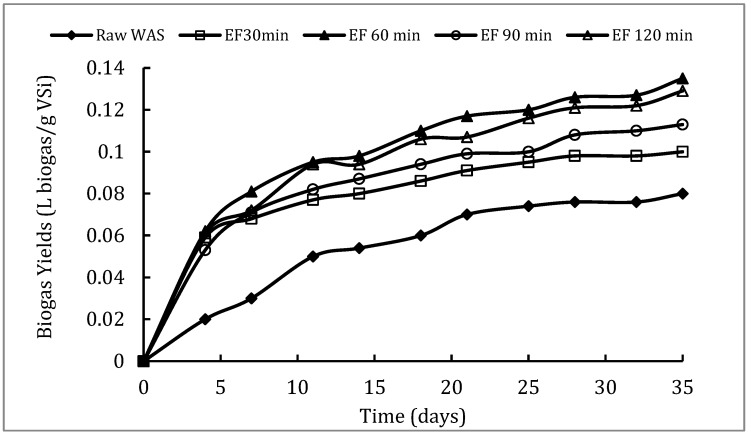
Biogas yields during batch anaerobic fermentation of raw and EF-pretreated (0, 30, 60, 90, and 120 min) sludge.

To determine the effect of EF pretreatment time on anaerobic biodegradability and to optimize pretreatment time, batch anaerobic fermentations were performed. WAS samples pretreated by EF at different times (0, 30, 60, 90 and 120 min) were thus used as substrates. Figure 3 provides the kinetics of cumulative biogas yields obtained during these fermentations. In comparison to raw sludge fermentation, no time lag for biogas production was observed at the beginning of pretreated sludge fermentations. A biogas yield of about 0.080 L biogas/g vs. was achieved with raw WAS. However, an increase in biogas yields was observed for all pretreated samples in comparison to raw samples. Thus, yields calculated at the end of fermentation were 0.100, 0.113, 0.135 and 0.129 L biogas/g VS, respectively, for sludge pretreated by EF during 30, 60, 90 and 120 min. 

### 2.2. Semi-Continuous Fermentation of Raw and Pretreated WAS

**Figure 4:**
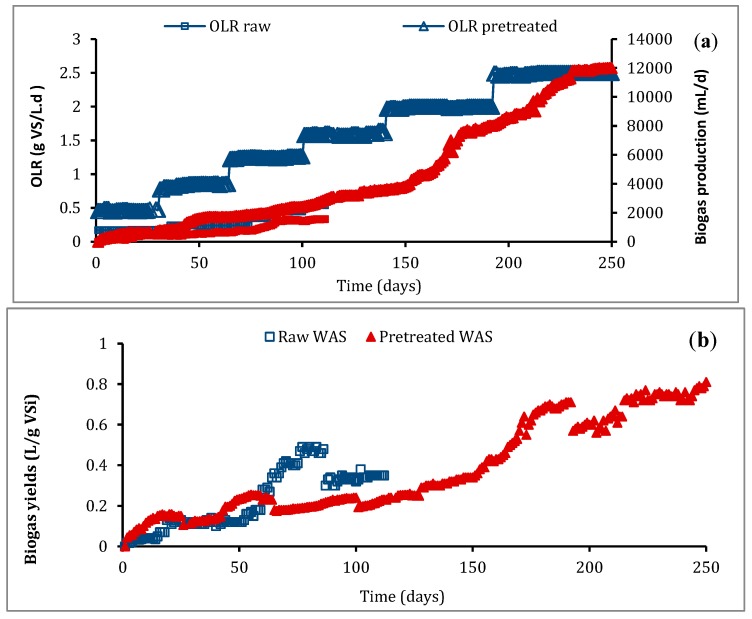
Evolution of organic loading rate (**a**) and biogas yield (**b**) during semi-continuous fermentation of raw and pretreated sludge in an up-flow anaerobic sludge blanket reactor.

In a first step of this study, the digester was fed with raw sludge at an organic loading rate (OLR) increasing from 0.17 g VS/L.d to 0.54 g VS/L.d (day 130). For the pretreated sludge, the first OLR applied to the reactor was 0.45 g VS/L.d. This parameter was then gradually increased to 2.5 g VS/L.d (Figure 4a). During these two fermentations, different HRTs (20, 14, 10 and 7 days) were applied which decreased with increasing OLR.

According to data presented in Figure 4a, the daily biogas production in the two cases was low during the first days of fermentation and then increased with the increase of OLR. Figure 4b provides the biogas yields for raw and pretreated sludge. Low biogas yields were observed during the first 20 days: 0.03 and 0.14 L/g VS, respectively, in the case of raw and pretreated sludge. Improvement in the biogas yield was observed after 20 days of feeding which represent the reactor HRT. Indeed, maximum daily biogas production rates of about 1.56 and 12 L/d were recorded during the fermentation of raw and pretreated sludge, respectively, which corresponds to a biogas yield of about 0.35 and 0.81 L/g vs. (Figure 4b).

**Figure 5:**
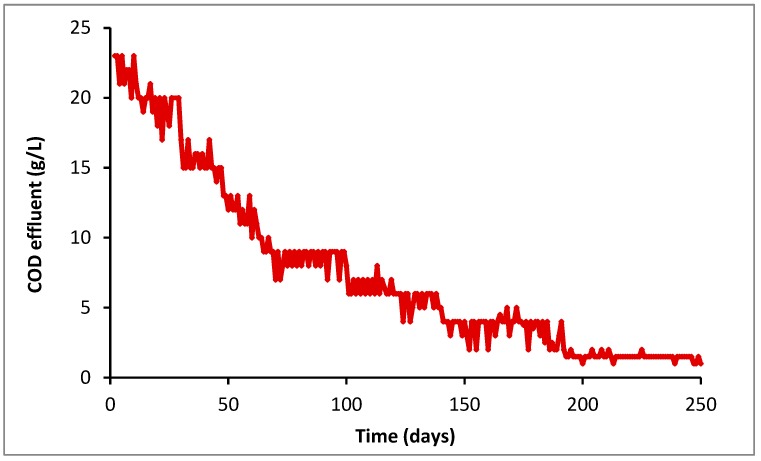
Evolution of total COD of effluent from UASB reactor digesting EF-pretreated sludge.

The reactor effluent COD was determined during this study. Figure 5 illustrates the evolution of this COD effluent during the fermentation of EF-pretreated sludge. When fermentation began, the COD effluent was very high. This confirms the low degradation of raw sludge during the fermentation study of raw sludge when the COD values were around 20 g/L (data not shown). The total COD of pretreated sludge was observed to be about 26 g/L. This value then decreased after passing through the bed sludge reactor. COD removal was enhanced during the fermentation step, reaching 96.7% at an OLR of 2.5 g VS/L.d.

## 3. Discussion

The physicochemical characteristics of WAS before and after EF pretreatment, provided in Table 1, point to the improvement of sludge quality after subsequent anaerobic treatments, both in terms of pH and organic matter solubilization (COD, VFA). On one hand, the increase of pH can be explained by the generation of hydroxyl radicals by electrochemical reactions and by the decrease in H^+^ production [21]. On the other hand, after 2 h of treatment, residual flocs precipitated with iron, which was continuously dissolved from the iron anode as governed by the law of Faraday. This observation has also been made for industrial effluents treated with an electro-coagulation process using a cast iron anode [22]. The increase in conductivity was probably related to the release of mineral salts during cell lysis but also to the mineralization of organic matter [23]. According to COD analyses, a solubilization reaction occurred during the pretreatment. However, the decrease of total COD during the second hour of treatment can be explained by the mineralization of organic matter due to the oxidation of simple molecules released into the solution. The release of VFA (acetic acid, isobutyric acid, propionic acid) was also observed in correlation with a soluble COD increase which probably results from the solubilization of the cellular content during the treatment [24]. This increase was higher than that obtained by Xu et al. [25] who found a total VFA concentration of 396.2 mg/L after 40 min of electrochemical sludge treatment (Ti/RuO_2_ anode) relative to an initial concentration in raw sludge of 86.7 mg/L. Therefore, the presence of VFA in pre-treated sludge should obviously promote the subsequent anaerobic digestion process.

Examination of WAS dewaterability pointed out that EF tends to decrease CST values of pretreated samples as a function of treatment time. Theoretically, high CST values indicate a low dehydration rate, whereas low CST values indicate greater dehydration properties [26]. Consequently, EF reactions considerably reduce the required time for dewatering sludge. This implies that dissociation of sludge flocs had occurred. The decrease in the biosolid resistance to dewatering in terms of CST was also observed with Fenton pretreatment of biological sludge [27]. According to Jin et al. [28], proteins and polysaccharides, which are the main constituents of EPS, actively contribute to the water retention capacity in sludge flocs. It is therefore conceivable that material released following the disruption of the cell wall increases the available surface area of these compounds [28]. Many previous studies have also demonstrated how decantation, bioflocculation, and sludge dewatering show an excellent relationship with the EPS content and EPS spatial distribution [29,30]. Liu et al. [31] and Xu et al. [32] reported that the disruption of flocs and cells by Fenton pretreatment led to the solubilization of EPS and acceleration of solid–water separation.

The FTIR method is based on the absorption of infrared radiation by the analyzed material. It allows for both the detection of the characteristic vibrations of the chemical bonds and the analysis of the chemical functions present in the material [31]. Infrared spectrophotometer analysis in the 400–4000 cm^−1^ range is most often performed because this is where most of the frequencies of the functional groups are located [32]. Relevant peaks attributed to special functional groups according to the literature are summarized in Table 2. 

According to the results, the FTIR spectrum of pretreated sludge presented a change in the intensity and shift of peaks in comparison to the raw sample. The strong peak around 3100–3500 cm^−1^ observed in the two spectrums corresponded to NH and OH stretching vibrations including hydrogen bonds. The increase in intensity and the shift towards a lower frequency of this band in the spectrum of the treated sample reflect an increase in hydrogen bonds which justifies the production of proteins with amine functions (NH_2_). The rising intensity of the main bands after EF pretreatment can be explained by the increase in reducing sugar (C=O carboxyl functions) and protein (NH_2_ amine functions) concentrations which are released into the supernatant after cell lysis. The appearance of new peaks observed around 1100–950 cm^−1^ has been attributed to polysaccharides by Naumann et al. [33]. In the present case, these polysaccharides are released from exopolymers (EPS) dissociated during the disintegration of WAS. This can account for the improvement in dewaterability of pretreated samples. However, the apparent peak at 1030 cm^−1^ in the pretreated sludge spectrum is related to the vibration of the Si–O–Si function [32], due to the release of silicon organic compounds (siloxanes, silanols) present in the waste activated sludge. Siloxanes are generally adsorbed to EPS flocs because of their low solubility in water. These compounds have been detected in biogas during the AD of sludge, resulting from their release during organic matter degradation and the increase in temperature within the anaerobic digester [34]. Indeed, their presence in the pretreated sludge can be explained by EPS dissociation during the disintegration of sludge by EF reaction. The 2800–1900 cm^−1^ range is related to elongations of the triple bonds C≡C and C≡N (acetylene, cyanogen) and cumulative double bonds X=Y=Z [32]. Compared with the raw WAS spectrum, this range presented vibrations at 2015, 2176, and 2359 cm^−1^ in the case of pretreated sludge. This could be due to the effect of EF on the transformation of certain molecules into molecules with triple or double bonds.

Batch anaerobic fermentations of raw and pretreated samples performed in similar conditions led to an improvement in the biogas yield after EF pretreatment. The low biogas yield of raw WAS characterizes its low biodegradability as well as the rate-limiting stages of anaerobic digestion as hydrolysis due to the strong protection of EPS and cell walls. Therefore, as demonstrated in the present study with EF process application, a pretreatment step is crucial for biodegradability as well as the entire sludge AD performance to be enhanced [14]. Pretreated sample biogas yields have thus been improved in comparison with raw sludge. In addition, EF pretreatment allows for AD to be accelerated, since there is no more time lag for biogas production. Biogas improvement has been observed to correlate with treatment time. Indeed, a significant improvement in the biogas yield occurs after 60 min of pretreatment compared with the other treatment times. An increase in the biogas potential of about 68% was obtained, coinciding with the highest VFA concentration released during the treatment (Figure 1b). These results imply that EF pretreatment during 30 or 60 min has a positive effect on the anaerobic digestion process by increasing the biodegradability and biogas yield.

In order to comprehend the effect of EF pretreatment on the AD process, semi-continuous fermentations on raw and pretreated sludge were investigated in a lab-scale reactor. The low biogas yields observed during the first 20 days of both fermentations could be explained by the absence of degradable substrates in the upper part of the digester which concerns methanogens. Comparison between results highlighted how the biogas yields improved when EF-pretreated sludge was used as feed for the reactor, thus confirming the batch test results. A 2.3-fold increase in biogas yields was achieved. Table 3 summarizes the results from both fermentations and also indicates the improvement in the methane percentage of biogas during the fermentation of EF-pretreated sludge that reached 68% against 56% in the case of raw sludge fermentation.

In addition, a strong COD removal rate (96.7%) was measured in the effluent even at low HRT (7 days) and at an OLR of 2.5 g VS/L.d. This improvement in COD removal could result from the increase in sludge biodegradability and from the purification performance of the Up-flow anaerobic sludge blanket reactor (UASB).This performance is higher than those obtained by Xu et al. [35] and Li et al. [36] who reported a COD removal of about 49.2% and 12.5%, respectively. Yuan et al. [37] also demonstrated that a combination of electrochemical and sodium hypochlorite pretreatments significantly enhanced the biogas yields by about 1.83-fold the sludge anaerobic digestion and shortened the stabilization period. According to Li et al. [38] and Chong et al. [13], the anaerobic UASB reactor is the most robust digester for the sludge treatment. Indeed, this type of reactor contains microorganisms that form granules through which, in an upward movement, the distributed effluent passes through the base of the reactor [39]. Hence, the proposed sludge treatment system within the UASB can potentially improve treatment efficiency. It can also reduce the discharge of pollutants into the environment as well as reduce the sludge toxicity in aquatic environments [40,41].

All these results confirm the efficiency of EF sludge pretreatment for improving biogas production. This technique therefore allows for sludge to become more accessible to the anaerobic consortium, consequently enhancing the anaerobic process and biogas yield. In addition, the UASB system has proven its effectiveness by significantly improving sludge anaerobic degradation, since the different microbial communities presented in the digester are probably well balanced.

## 4. Materials and Methods

### 4.1. WAS and Anaerobic Inoculum

The WAS sample was obtained from a municipal wastewater treatment plant (WWTP) located in the Sidi Mansour region (north of Sfax, Tunisia) with a capacity of 17,900 m^3^/d. Collected samples were stored at 4 °C for subsequent experiments. The characteristics of the WAS sample are provided in Table 1. For anaerobic digestion experiments (batch and semi-continuous fermentations), the inoculum was sampled from a semi-pilot anaerobic bioreactor installed in the laboratory.

### 4.2. Electro-Fenton Pretreatment

Electro-Fenton treatment was carried out under optimum conditions determined during a previous study [42]. The reaction took place in a 500 mL glass reactor using an ASF type 400/40.10 electric generator to apply a current density of 2.5 A/dm^2^. It comprised one pair of anodic and cathodic electrodes (cast iron plates) which were positioned approximately 2 cm apart from each other and were dipped in the effluent. The total effective surface area of electrodes was 0.16 dm^2^. During this treatment, the main electrochemical reactions occurred at the anode with oxidation of iron and at the cathode with the reduction of water. It was based on the principle of soluble iron anodes. Iron electrodes were used for in-situ generation of Fe^2+^. In each run, 300 mL of raw WAS were treated and operated in batch mode. The pH of the WAS sample was adjusted to 3 by adding an HCl solution (2N). The H_2_O_2_ (30% *v*/*v*) was added after adjustment of pH to the desired value; the pH of the solution was not controlled again during the reaction. The aqueous solution of reactants was homogenized by magnetic agitation to avoid sedimentation of WAS particles and produced at room temperature.

### 4.3. Batch Anaerobic Digestion

Batch AD tests were conducted to determine the effect of EF pretreatment time on biogas production. WAS samples pretreated by EF at different times (0, 30, 60, 90 and 120 min) were used as substrates. For each treatment time, three batch reactors with a capacity of 120 mL were tested in parallel. Batch reactors treating raw sludge were also conducted for comparison. In each reactor, substrate and inoculum were introduced with a vs. substrate/VS inoculum ratio equal to 1. All batches were adjusted to 7.2 then purged with a gas mixture of 75% N_2_ and 25% CO_2_ for 3 min to maintain anaerobic conditions and finally incubated under mesophilic conditions (37 ± 1 °C) for 35 days. During fermentation, biogas production was measured using a gas displacement device. The Biochemical methane potential (BMP) experiments and the related analysis were performed by the Bio2E platform [43].

### 4.4. Semi-Continuous Anaerobic Reactor

An up-flow anaerobic sludge blanket (UASB) reactor was used to study the semi-continuous fermentation of WAS before and after 1 h of EF pretreatment. The working volume of the digester was 7 L. For maintaining a constant temperature (37 °C), it had a PVC double wall filled with heated water from a heated bath circulator. Before beginning the experiments, the bioreactor was inoculated with the anaerobic microbial consortium and fed with raw sludge (0.05 g VS/L.d) during 2 months. This preparation period ensured biomass enrichment and process stability. The hydraulic retention time (HRT) during fermentation of raw and pretreated sludge was set to 20 days during the first period. It was then reduced to 14, 10 and 7 days. Feeding and withdrawing were done once a day, using a pump. The volume of biogas was measured by liquid displacement. During the experiments, the COD of influent and effluent, biogas production, pH, and VFA were monitored.

### 4.5. Analytical Methods

The pH was measured with a pH meter (Metrohm). Total and soluble COD were quantified with a titration method after a total digestion with H_2_SO_4_ and potassium dichromate at 150 °C for 2 h [42]. BOD_5_ was determined by the manometric method with a respirometer (BSB-Controller Model 620 T (WTW)). Total solids (TS) and Total suspended solids (TSS) were measured by weighing samples before and after overnight drying at 105 °C. Volatile solids (VS) and volatile suspended solids (VSS) were analyzed by loss on ignition at 600 °C for 2 h. The total Kjeldahl nitrogen content (TKN) and the ammoniacal nitrogen (N–NH_4_^+^) were analyzed according to the Kjeldahl-N method.

Conductivity was measured using a conductimeter (CONSORT). Total volatile fatty acids (VFA) were analyzed by centrifuging the samples for 15 min at 8000 rpm and then filtering them on 0.45 μm pore size syringe filters. The resulting filtrates were acidified (pH 3) with HCl (0.1 N) before being analyzed and quantified by high-performance liquid chromatography (HPLC: SHIMADZU 10 AVP). Fourier transform infrared spectroscopy (FTIR 380 Nicolet model) equipped with a He-Ne laser and a telluride, mercury, and cadmium detector (MCT) at a frequency of 400–4000 cm^−1^ was used to analyze the organic functional groups present in raw and pretreated WAS. Before the FTIR analysis, sludge samples were centrifuged for 20 min at 4500 rpm and then filtered through a 0.45 μm pore size membrane filter.

The capillary suction time (CST) was measured with a CST analyzer (Triton Electronics Ltd., United Kingdom) using a 7 × 9 cm^2^ size CST paper. All sludge samples were used in their initial state without centrifugation and filtration. A volume of 5 mL of sample was placed into a metal tube and the ring time was recorded as CST.

## 5. Conclusions

The present study has highlighted the effect of EF pretreatment on the efficiency of disintegration and AD of sludge. The pretreatment has shown to improve organic matter solubilization by increasing soluble COD and volatile fatty acids. FTIR analysis also revealed the release of reducing sugars, polysaccharides, and proteins, thus confirming the dissociation of flocs. This result can account for the improvement in sludge filterability indicated by low CST results of 22.4 and 14 s after 1 and 2 h of pretreatment, respectively. Results from batch and semi-continuous anaerobic fermentations have confirmed the positive effect of the EF process in enhancing the biogas potential and stability of the anaerobic system. The EF process therefore promises to be a more reliable and robust solution for the enhancement of WAS anaerobic treatment.

## Figures and Tables

**Table 1:** Physicochemical characteristics of waste activated sludge (WAS) before and after pretreatment.

Parameters	Raw WAS	EF-Pretreated WAS
pH	6.95 ± 0.2	7.5 ± 0.8
Conductivity (mS/cm)	3.72 ± 0.3	13.72 ± 0.1
TS (g/L)	19.45 ± 1.4	14.28 ± 2
VS (g/L)	12.67 ± 1.2	10.34 ± 1.3
TSS (g/L)	15.16 ± 0.9	10.5 ± 0.5
VSS (g/L)	7.27 ± 1.3	3 ± 0.6
TCOD (g/L)	20.4 ± 4	26 ± 1.2
SCOD (g/L)	1.73 ± 2	4.1 ± 0.3
NTK (mg/L)	914 ± 10	920 ± 3
VFA (mg/L)	84.3 ± 13	925 ± 35

**Table 2:** Functional groups or compounds analyzed by Fourier transform infrared spectroscopy (FTIR) analysis in raw and EF-pretreated sludge.

Wave Number (cm^−1^)	Vibration and Functional Groups
1634	C=O carboxylic acids
	C=C alkenes
	OH adsorbed water
3275	O-H hydroxyl group and water
	NH_2_ amine

**Table 3:** Evolution of organic loading rate (OLR), biogas yield and methane percentage during anaerobic digestion of raw and pretreated sludge in UASB reactor.

Feed Sample	OLR (g VS/L.d)	Biogas Yield (mL/g VS)	CH_4_ (%)
Raw	0.25 ± 0.02	434 ± 0.017	48 ± 4
	0.35 ± 0.01	480 ± 0.031	54 ± 2
	0.50 ± 0.02	440 ± 0.024	56 ± 1
Pretreated	0.50 ± 0.016	400 ± 0.028	58 ± 3
	1.60 ± 0.018	625 ± 0.030	66 ± 2
	2 ± 0.027	735 ± 0.032	68 ± 1
	2.50 ± 0.030	685 ± 0.029	67 ± 3

## Abbreviations

AD	anaerobic digestion
COD	chemical oxygen demand
CST	capillary suction time
EF	electro-Fenton
FTIR	Fourier transform infrared spectroscopy
OLR	organic loading rate
VFA	volatile fatty acids
WAS	waste activated sludge
